# Predicting future brain atrophy based on longitudinal MRI

**DOI:** 10.1177/13872877261471049

**Published:** 2026-07-28

**Authors:** Maryam Hadji, Elaheh Moradi, Jussi Tohka

**Affiliations:** 1 A.I. Virtanen Institute for Molecular Sciences, University of Eastern Finland, Kuopio, Finland

**Keywords:** Alzheimer's disease, dementia, hippocampal atrophy, machine learning, mild cognitive impairment, MRI, progression prediction, total gray matter atrophy, ventricle enlargement

## Abstract

**Background:**

Neuron loss is a hallmark of neurodegenerative diseases and leads to brain atrophy detectable with magnetic resonance imaging (MRI). Accurate prediction of future atrophy is valuable for research in Alzheimer's disease (AD) and related dementias.

**Objective:**

This study aimed to predict annualized percentage changes in hippocampal, ventricular, and total gray matter (TGM) volumes in individuals ranging from cognitively normal to dementia, and to evaluate whether longitudinal MRI-derived change measures improve prediction performance compared with single-time-point MRI information.

**Methods:**

Using elastic net regression, we compared baseline models based on single-timepoint MRI information with longitudinal models incorporating prior MRI-derived change measures. Both approaches were evaluated as MRI-only and MRI + risk-factor variants, with risk factors including age, sex, *APOE4*, and diagnostic status.

**Results:**

In cross-validated analyses using the Alzheimer's Disease Neuroimaging Initiative (ADNI) cohort, the longitudinal MRI + risk-factor model performed best, yielding Pearson correlations of 0.62 for hippocampal atrophy, 0.51 for ventricular enlargement, and 0.41 for TGM atrophy. Longitudinal models consistently outperformed single time-point models, and adding risk factors improved predictive performance beyond MRI alone. External validation using the Australian Imaging, Biomarkers and Lifestyle cohort confirmed these findings. Predicted atrophy outperformed present-day regional volumes in identifying individuals progressing from normal cognition to MCI/dementia and from MCI to dementia.

**Conclusions:**

MRI-derived longitudinal features enhance atrophy prediction, and predicted atrophy rates provide sensitive markers of future cognitive decline. These findings support the potential utility of predicted atrophy for cohort enrichment and therapeutic trial design.

## Introduction

Dementia impacts approximately 57 million people globally,^1^ with Alzheimer's disease (AD) being its most common cause.^2^ Machine learning approaches for predicting future diagnoses have been extensively studied in the context of dementia,^3^ with numerous studies focusing on the progression from mild cognitive impairment (MCI) to dementia.^4^ However, as the clinical focus shifts from prognosis to selecting the most effective treatments, there is a growing need to predict more nuanced outcomes.^5,6^ This shift in focus could also enhance the selection of study participants for clinical trials, ensuring that the most appropriate candidates are chosen based on their predicted responses to treatment.

Predicting the change in cognitive tests scores, such as Mini-Mental State Examination (MMSE) and Alzheimer's Disease Assessment Scale–Cognitive Subscale (ADAS-Cog), is perhaps the most natural way towards these more nuanced outcomes. However, predicting changes in cognitive scores has proven to be challenging, partly due to the validity challenges in the longitudinal measurement of cognitive changes in less impaired populations with the well-established tests^7,8^ and partly due to challenges in assessing cognition in general.^9^ These challenges include learning effects, which can skew results due to repeated testing, and high variability, where outcomes depend significantly on the individual's condition on the test day. For example, in the recent TADPOLE challenge, none of the competing teams was able to perform better than a simple baseline method for the ADAS13 score prediction while the predictions for the future diagnosis and future ventricular volume were significantly better than with the baseline methods.^10^ Moreover, while the future cognitive scores may be predictable, this predictive power is many times driven by the current baseline scores, yielding change estimates of limited usability.^11^ As a result, there is a pressing need for serial biomarkers that reflect the underlying biology of AD and its connection to dementia.

This work, instead, explores the prediction of future atrophy based on serial magnetic resonance imaging (MRI). The MRI-based brain atrophy has long been identified as an important measure to track dementia progression.^12^ It possesses several important characteristics for a prediction target: 1) atrophy measures are quantitative, 2) MRI is widely available and non-invasive, 3) various atrophy measures have been adopted as secondary endpoints in randomized controlled trials of dementia/AD interventions^13,14^ and 4) hippocampal atrophy has been qualified for enrichment in pre-dementia trials by the European Medicines Agency.^15^ By focusing on atrophy prediction, this study aims to provide a more reliable and clinically useful tool for both treatment selection and trial participant selection.

We focus on three distinct MRI biomarkers of neurodegeneration: hippocampal atrophy, ventricular enlargement, and total gray matter (TGM) atrophy, which are associated with AD progression.^16–19^ These measures are widely used in clinical studies and have also been included as secondary endpoints in dementia and AD trials,^20,21^ where longitudinal changes in brain volumes are used to assess disease progression and to explore potential treatment effects. Changes in these structures have been linked to cognitive decline and clinical progression.^22–24^ In this study, TGM atrophy was used as a proxy for whole-brain atrophy, as gray matter degeneration is closely related to neuronal loss and cognitive decline in AD.

Surprisingly, while the association of brain atrophy measures to dementia and AD progression is well documented, the prediction of future changes in these measures has not been studied widely. Prediction of the future ventricle volume was one of the tasks in a recent TADPOLE challenge,^10^ where the best performing model was based on the concept of rate of AD progression.^25^ Liedes et al.^26^ focused on prediction of hippocampal atrophy with promising results. However, in Liedes et al.,^26^ they studied only prediction of hippocampal atrophy and used data from single time-points only for predictions. In this work, we analyze three regions (hippocampus, total gray matter, and ventricles) together within the same dataset and workflow and develop a modeling framework able to use predictors derived from longitudinal data. Finally, we study the value of forecasted atrophy rates to predict the progression of the level of the memory concern from healthy to MCI/dementia and from MCI to dementia. We demonstrate that the models that use predictors derived from longitudinal data consistently outperform corresponding models based on single time-point predictors. Also, we show that predicted atrophy is a better indicator of progression to mild cognitive impairment and dementia than the volumes of the regions at current day. The primary aim of this study was to predict future annualized structural brain changes, including hippocampal atrophy, ventricular enlargement, and TGM atrophy, using MRI-derived imaging features and demographic and risk-factors. We specifically compared: baseline models based on single-time-point MRI information with longitudinal models incorporating prior MRI-derived change measures, and MRI-only models with models combining MRI and clinical and risk-factor variables. We hypothesized that longitudinal MRI-derived predictors would improve prediction performance compared with single-time-point predictors and that predicted future atrophy rates would provide more sensitive indicators of future cognitive progression than present-day regional brain volumes alone.

## Methods

### Data for atrophy/enlargement prediction

Data used in this work were obtained from the Alzheimer's Disease Neuroimaging Initiative (ADNI) (http://adni.loni.usc.edu) and the Australian Imaging, Biomarker and Lifestyle (AIBL) study group (https://aibl.org.au). The ADNI was launched in 2003 as a public-private partnership, led by Principal Investigator Michael W. Weiner, MD. The primary goal has been to test whether serial MRI, PET, other biological markers, and clinical and neuropsychological assessment can be combined to measure the progression of MCI and early AD. For up-to-date information, see (www.adni-info.org). AIBL study methodology has been reported previously.^27^

We included all ADNI participants who had a T1-weighted MRI acquired at the time of entry, 24-month visit, and 48-month visit. Moreover, we required participants to have information on demographics (age, sex), *APOE4* status, and diagnosis status obtained from from the ADNIMERGE.csv table. 2365 participants had MRI data at the time of entry. After 24 months of follow-up, MRI information was available for 1373 participants, and for 586 participants after 48 months. 565 individuals had MRI data at entry, 24 months, and 48 months. After excluding participants without *APOE4* information, we finalized our dataset with 550 individuals who had all the considered information. We refer to this dataset as ADNI-main dataset. The ADNI-main cohort included 550 participants with MRI data available at baseline, 24-month, and 48-month visits. The external AIBL cohort included participants with MRI data available at baseline, 18-month, and 36-month visits. Thus, each participant contributed three longitudinal MRI visits spanning approximately four years in ADNI and three years in AIBL. The annualized structural change measures were estimated between predefined study visits (ADNI: baseline, 24-month, and 48-month visits; AIBL: baseline, 18-month, and 36-month visits). Participants without the required MRI visits or demographic/risk-factor information were excluded from the analyses. Therefore, only participants with complete data at the predefined time points were included, and missing visits or dropout were handled through this inclusion criterion.

We used the Australian Imaging Biomarkers and Lifestyle Flagship Study of Aging (AIBL, ADNIsubcohort) as an external test data in our study. At the study entry, MRI scans were available for 605 participants. After 18 months of follow-up, 232 individuals had MRI scans, and 149 individuals had scans at the 36-month follow-up. Among them, 95 participants had MRI scans at all three time points. All these 95 individuals have complete demographic data, including age, sex, diagnosis, and *APOE4* status. The characteristics of study cohorts are presented in Table 1 and participant's RIDs are available in the Supplemental Material.

**Table 1.** Characteristics of the ADNI and AIBL cohorts, including sample size, age, sex distribution, *APOE4* status, and clinical diagnosis. Age is presented as mean (standard deviation). Diagnosis and age are from the time of entry. CN refers to cognitively normal. *APOE4* status is displayed as the count of individuals with 0, 1, or 2 alleles.

**Baseline characteristics**	**ADNI**	**AIBL**
Sample size, N	550	95
Age, years	73.04 (6.07)	73.31 (6.93)
Sex, M/F	297 / 253	48 / 47
*APOE4*, 0/1/2	321 / 186 / 43	60 / 29 / 6
Diagnosis, CN/MCI/AD	246 / 236 / 68	76 12 / 7

### MRI preprocessing

The MRI data were preprocessed following the framework described in.^28^ We used the volumetric and thickness measures computed with CAT12 toolbox (https://neuro-jena.github.io/cat/, version 8.1) using the default settings^29^: For 136 volume measures, the T1-weighted MRI was segmented into gray matter (GM) and white matter (WM) and non-linearly normalized to a stereotactic space using the shooting approach.^30^ Following Karaman et al.,^31^ we included intracranial volume as one of the MRI features, rather than serving solely as a normalization factor, alongside regional volumetric measures. Based on spatially normalized GM and WM segments, the neuromorphometrics atlas was used to extract the regional volumes. The atlas is derived from the maximum probability tissue labels derived from the “MICCAI 2012 Grand Challenge and Workshop on Multi-Atlas Labeling” (http://www.neuromorphometrics.com/2012) MICCAI Challenge Data.htm with the MRIs from OASIS project and the labeled data provided by Neuromorphometrics, Inc. (Neuromorphometrics.com) under academic subscription. We denote these volume measures by *V_ij_*(*t*) for participant *i*, region *j* = 1*,…,*136, and time point *t*. The cortical thickness measures^32^ were computed and registered to the FSaverage surface template,^33^ where DK40 atlas^34^ was used to define 69 regionally averaged cortical thickness measurements. These cortical thickness measures are denoted by *V_ij_*(*t*) for participant *i*, region *j* = 137*…,*206, and time point *t*. The MRI data from the participant *i* at time point *t* is collected into a vector *V_i_*(*t*). The cortical thickness and regional volumetric measures were used as predictor variables in the machine learning models. However, the primary prediction targets in this study were hippocampal atrophy, ventricular enlargement, and TGM atrophy.

The annual percentage change between the time points *t*_1_ and *t*_2_ (expressed in years) for a region *j* is defined as

(1) $$\Delta_{ij}(t_{1},t_{2})=\frac{V_{ij}(t_{1})-V_{ij}(t_{2})}{V_{ij}(t_{1})(t_{2}-t_{1})}.$$

If Δ*_ij_* (*t*_1_*,t*_2_) is positive, it refers to atrophy, and if Δ*_ij_* (*t*_1_*,t*_2_) is negative, it refers to enlargement. Thus, the prediction targets in this study correspond to annualized percentage change in MRI-derived structural brain measures.

### Prediction framework

Our purpose is to predict future Δ*_ik_* (*t*_2_*,t*_3_) based on the data available during time points *t*_1_ and *t*_2_ where *t*_3_ *>* *t*_2_ *>* *t*_1_. The prediction task focused on forecasting future structural brain changes between the current-day and future MRI visits using information available from previous MRI assessments and demographic/risk-factor variables. The prediction task was designed to evaluate whether incorporating longitudinal MRI-derived information improves prediction of future structural brain changes compared with models based only on current MRI measurements. In addition, we evaluated whether the predicted future atrophy rates provide clinically meaningful information for subsequent progression prediction. For simplifying the terminology, we name the time-point *t*_1_ as past, *t*_2_ as current day, and *t*_3_ as future. We formulate this prediction as supervised learning problem. Denote future change Δ*_ik_* (*t*_2_*,t*_3_) by *y_ik_* and the number of participants by *N*. Provided the data **x***_i_,i* = 1*,…,N* (see Table 2) from the past and current day, we apply Elastic Net linear regression, a regularized linear regression method that combines both Lasso (L1) and Ridge penalties (L2).^35^ In Elastic Net regression, the regularization parameter *λ* controls the overall strength of penalization, while the parameter *α* determines the balance between L1 and L2 penalties. Elastic Net is advantageous in high-dimensional data environments, especially when multicollinearity (correlation among predictors) is present. This method allows us to improve model generalization by reducing over-fitting while selecting a sparse subset of informative features. Dropping the index *k*, elastic net solves the coefficients $\hat{\beta }$ of the linear model as

$$\begin{array}{rl} \hat{\beta } & =\min\;\arg\;(\beta )\sum_{i=1}^{n}{\left(y_{i}-x_{i}\beta \right)}^{2}\\ & +\lambda \left(\alpha \sum_{\;j=1}^{p}|\beta_{j}|+(1-\alpha )\sum_{\;j=1}^{p}\beta_{j}^{2}\right) \end{array}$$

where *β* = [*β*_1_*,…,β_p_*]*^T^*, *λ* controls the overall regularization strength, and *α* adjusts the balance between L1 and L2 penalties. When *α* = 1, Elastic Net behaves like Lasso regression, driving some coefficients (*β_j_*) to exactly zero, effectively selecting relevant features. Conversely, when *α* = 0, it acts as Ridge regression, reducing the effect of multicollinearity without eliminating predictors. By setting 0 *<* *α* *<* 1, Elastic Net captures the advantages of both Lasso and Ridge regularization, yielding a stable and interpretable model even in the presence of correlated predictors.

**Table 2.** Definitions of the predictive models used in the study. The models vary by the inclusion of MRI data, MRI changes over time, and additional risk factors such as age, sex, diagnosis, and *APOE4* status.

Model	Predictors	x*_i_*
Baseline, MRI only	Current day MRI	*V_i_*(*t*_2_)*,***f***_i_*
Baseline, MRI + Risk factors	Current day MRI, age, sex, diagnosis, APOE4	*V_i_*(*t*_2_)*,*Δ*_i_* (*t*_1_*,t*_2_)*,***f***_i_,***r***_i_*
Longitudinal, MRI only	Current day MRI, MRI change between the past and the current day	*V_i_*(*t*_2_)*,*Δ*_i_* (*t*_1_*,t*_2_)*,***f***_i_*
Longitudinal, MRI + Risk factors	Current day MRI, MRI change between the past and the current day, age, sex, diagnosis, APOE4	*V_i_*(*t*_2_)*,*Δ*_i_* (*t*_1_*,t*_2_)*,***f***_i_,***r***_i_*

We used (nested) cross-validation to optimize the parameter *λ*, ensuring that the selected model provided the best trade-off between model complexity and predictive accuracy. The parameter *α* was fixed at 0.5 following our previous works.^36^ This approach allowed us to obtain a model that is both interpretable and well-suited for generalization to new data.

We performed prediction for three primary structural MRI biomarkers: (1) hippocampal atrophy, defined as the annualized change in the combined left and right hippocampal volumes; (2) ventricular enlargement, defined as the annualized change in the combined left and right lateral and inferior lateral ventricular volumes; and (3) TGM atrophy, defined as the annualized change in total gray matter volume and used as a proxy measure of whole-brain neurodegeneration. Hippocampus and ventricles were defined according to the neuromorphometrics atlas (https://neuromorphometrics. com/Seg/html/segmentation/) and the TGM volume was defined as in the CAT12 toolbox.^29^

We designed four models based on two approaches with different feature combinations, presented in Table 2. In the baseline approach, in which we used current day data (ADNI 24 month visits) to predict atrophy in the future (ADNI 48 month visits), we defined two models: the MRI only model was trained using only MRI information and the MRI and risk factors model used in addition demographic/risk factor information containing age, sex, the number of *APOE4* alleles, and the current day diagnosis (normal cognition, MCI, dementia). Age, sex, diagnosis status, and *APOE4* were included because they are well-established factors associated with AD progression and structural neurodegeneration. We represented this demographic/risk-factor information by **r***_i_* for participant *i*. The longitudinal approach used past and current day data (ADNI 0 and 24 month visits) to predict atrophy in the future (ADNI 48 month visits), and we again defined MRI only and MRI + risk factors models. Since the MRIs were acquired both with 1.5 T and 3 T scanners, we provided this information to the learning algorithm as additional predictors (see^37^), encoding the field-strength combination for participant *i* as a binary vector **f***_i_*. The predictor variables used in the models included MRI-derived regional volumetric measures, cortical thickness measures, longitudinal MRI-derived change measures between previous and current visits (for longitudinal models), demographic and risk-factor variables including age, sex, diagnosis status, and *APOE4* allele count, as well as MRI scanner field-strength variables.

### Implementation and performance evaluation

**Figure 1.**
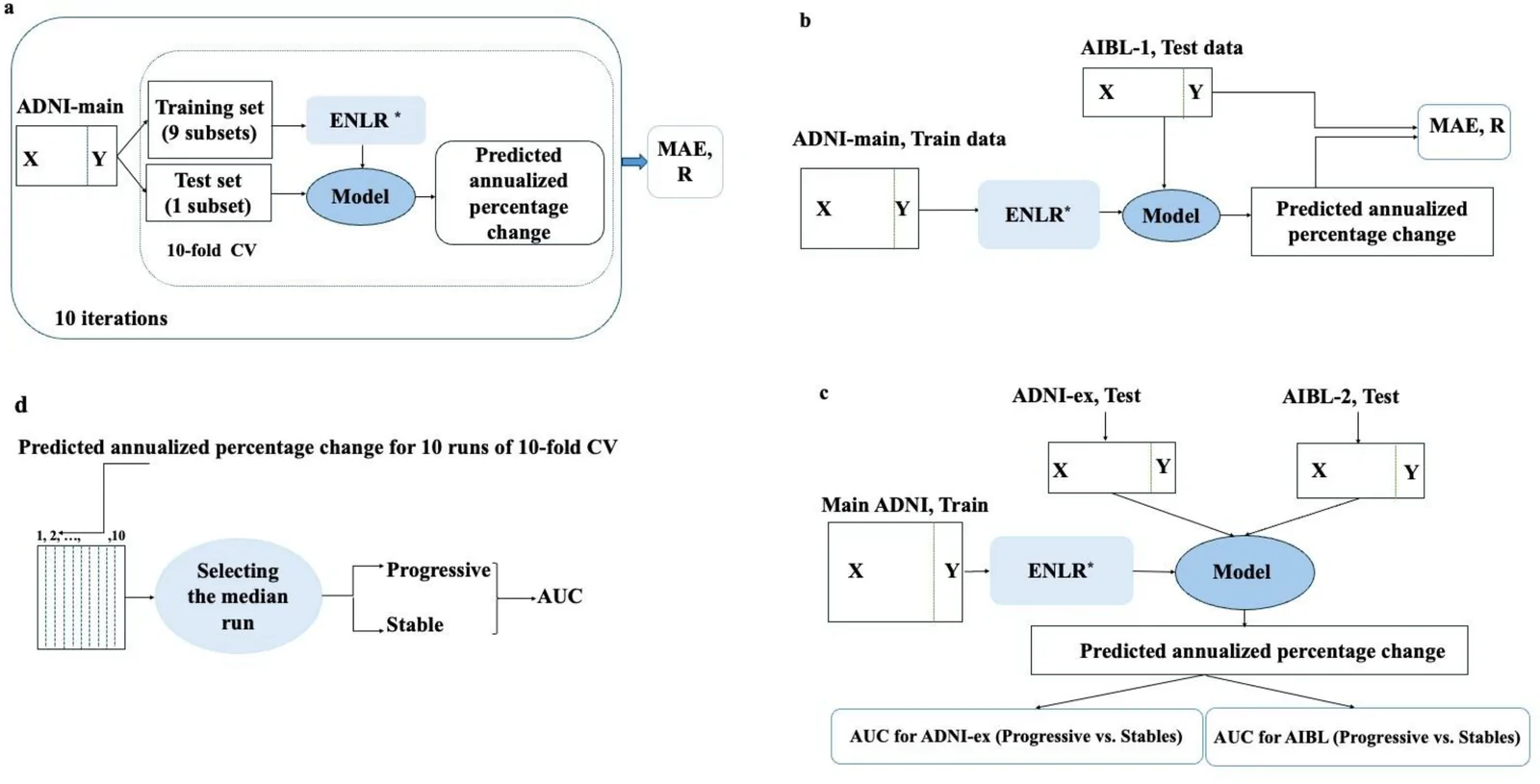
Schematic representation of the prediction framework. (a) Atrophy prediction framework: The model is trained and validated using a 10-fold cross-validation approach on the ADNI-main dataset. Elastic Net Linear Regression (ELNR) is used to predict the annualized percentage change. The performance is evaluated using Mean Absolute Error (MAE) and Pearson correlation (R). (b) External validation: The trained model is applied to the AIBL-1 test dataset (Table 1) predict the annualized percentage change, and the performance is assessed using MAE and Pearson correlation (R). (c) Progression prediction: The model is trained on the ADNI-main dataset and applied to external test datasets (ADNIexternal and AIBL-2, Table 3) to predict clinical progression. The Area Under the Curve (AUC) is used to evaluate classification performance for progressive vs. stable cases. (d) Selection of the representative run: The median run is selected from 10 iterations of 10-fold cross-validation, and participants are classified into progressive or stable groups based on predicted values. AUC is then calculated for the classification task. We use AIBL-1 and AIBL-2 to refer to two different versions of the AIBL test data, described in Tables 1 and 3, respectively.

Following the framework described in,^38^ we implemented a nested and stratified 10-fold crossvalidation (CV) framework to prevent overfitting and evaluate model performance, see Figure 1.

**Table 3.** Demographic and clinical characteristics of cohort used for validation of clinical progression prediction. The cohorts include ADNI-Main, AIBL, and ADNI-External datasets, with subgroups categorized as CN Progressive, CN Stable, MCI Progressive, and MCI Stable. Age is presented as mean (standard deviation). Sex is shown as M/F, and *APOE4* status is displayed as the count of individuals with 0, 1, or 2 alleles.

**Datasets**	**Group**	**N**	**Age, years**	**Sex, M/F**	***APOE4* (0/1/2)**
**ADNI-Main**	CN Progressive CN	60	73.74(6.63)	32/28	51/ 8/ 1
	Stable MCI	133	74.71(5.34)	64/69	85/46/2
	Progressive	79	71.90(7.24)	44/35	53/24/2
	MCI Stable	119	72.64(7.15)	71/48	56/46/17
**AIBL**	CN Progressive	13	74.38(6.56)	10 /3	4/7/2
	CN Stable	45	73.51(7.07)	16/29	36/8/1
**ADNI-External**	CN Progressive CN	25	75.42(5.27)	18/7	13/12/0
	Stable	56	69.69(5.74)	21/ 35	42/13/ 1
	MCIProgressive	46	73.81(6.78)	29/17	23/17/6
	MCI Stable	43	71.08 (7.56)	24/19	30/13/0

Stratification was performed based on the response variable to preserve its distribution across folds.^39^ The data splits were performed at the participant level, as each participant contributed a single prediction sample. The nested CV consisted of two levels: an outer loop for model evaluation and an inner loop for hyperparameter selection (*λ*). Participants were randomly divided into 10 subsets. In each fold of the outer loop, one subset was used for testing, and the remaining nine subsets were used for training. During the inner loop, the optimal *λ* was selected by minimizing the mean squared error (MSE), following the standard implementation of Elastic Net regression in the glmnet framework.^40^ All model training procedures, including feature scaling and hyperparameter optimization, were performed using only the training data within each CV loop and subsequently applied to the held-out test fold. No imputation or feature selection outside the Elastic Net regularization framework was performed. This nested approach ensures robust model evaluation and minimizes overfitting by effectively separating training and test data throughout the analysis pipeline.

We assessed the models’ performance using the cross-validated Pearson correlation coefficient (PCC) and the mean absolute error (MAE) between the predicted and actual annualized structural brain changes (atrophy/enlargement rates), expressed as annualized percentage change. While MSE was used internally for hyperparameter selection within the glmnet framework, PCC and MAE were used as the primary evaluation metrics because they provide complementary information regarding association strength and prediction accuracy.

To ensure robustness and minimize the influence of random variations, the results were averaged over 10 runs of nested 10-fold CV. To compare the correlation coefficients between the two approaches, we tested the results of a computation run with median PCC (6th largest PCC among 10 runs) using Hittner-modification of the Dunn-Clark test^41,42^ implemented in the CoCor package.^43^ Hittner modification of the test maintains better the type I error rate compared to the original Dunn-Clarke test for overlapping correlations. We computed the confidence intervals using a bootstrap procedure specifically designed for repeated CV.^44^ We considered all the comparisons planned and therefore report the results of all comparisons without adjustments for multiple testing.

Data in the ADNI originated from different phases of ADNI (ADNI1, ADNI2, ADNI3, ADNIGO) with different MRI protocols and scanner field strengths (1.5 T or 3.0 T). Our baseline approach to correct for differences in MRI markers due to scanner field strength was to include this information in the prediction model as explained in Section 2.3. This information was included as predictor variables to reduce potential variability related to acquisition differences across scanners and ADNI phases. Additionally, we tested ComBat to harmonize MRI data. ComBat, an empirical Bayes method for batch correction in microarray expression data,^45^ has been successfully utilized for harmonization in MRI.^46^ However, the addition of a harmonization step using the ComBat approach did not improve the prediction performance compared to our baseline approach (Supplemental Table 1).

We performed only minimal quality control of the segmentations. We visually inspected the scans with biologically implausible hippocampal atrophy rates with annual volume increase above 2%. The visual inspection indicated poor quality registration or segmentation. This quality control process led to exclusion of the data from two participants from the final analysis.

We further utilized the AIBL-cohort as an independent test dataset. The ADNI data served as the training set, while AIBL data was used for testing. The visit schedule of AIBL is different from the ADNI visit schedule: the current day are 18 (AIBL) and 24 (ADNI) month visit, and future are 36 (AIBL) and 48 (ADNI) month visit. However, because the prediction task was formulated as prediction of annualized percentage change, differences in follow-up duration between ADNI and AIBL cohorts were inherently accounted for in the outcome definition. All the analyses were done using R (version 4.1.1), with the following packages: glmnet,^40^ caret,^47^ cocor,^48^ pROC,^49^ ggplot2,^50^ and complexheatmap.^51^ The codes to reproduce the work are available at https://github.com/MaryamHadji/Future-prediction

### MCI/dementia progression prediction

We evaluated the effectiveness of our estimated atrophy/enlargement rates in predicting dementia progression (Figure 1(c), (d)). This analysis included participants from the CN and MCI groups. We predicted their future atrophy/enlargement rates and assessed how well these estimates predicted later changes in cognitive status, specifically CN-to-MCI/dementia or MCI-to-dementia progressions.

Model's performance was measured using the area under the receiver operating characteristic curve (AUC), and we considered each region of interest (hippocampus, ventricles, and TGM) separately. We divided participants within the CN and MCI groups into two categories based on their diagnosis at the current visit and follow-up data: 1) Stable group: CN/MCI individuals who have remained with the same diagnosis status for five years or more following the current day visit; 2) Progressive group: a) Individuals with CN diagnosis at the current day visit who later develop MCI or dementia, and b) MCI individuals who later develop to dementia . We then compared the ability of predicted future atrophy/enlargement rates to MCI/dementia progression prediction against a baseline using presentday brain region volumes. We hypothesized that predicted atrophy/enlargement rates better reflect future MCI/dementia progression than present-day brain region volume.

We used three different datasets for MCI/Dementia progression prediction: First, we used the main ADNI dataset, which we had previously used for atrophy prediction, comprising data from 550 individuals. Among the 246 CN individuals, 60 individuals progressed MCI/dementia during the followup period, and 133 remained stable with a follow-up duration of at least five years. Similarly, in the MCI group, 79 individuals converted to dementia, whereas 119 remained with MCI status, see Table 3. In this dataset, atrophy/enlargement rate predictions were generated within a cross-validation framework. For MCI/dementia progression prediction, we selected results from the cross-validation run with the median PCC and used the predicted atrophy/enlargement rates from that run to predict MCI/dementia progression.

Second, we trained our models to predict atrophy/enlargement rates $\Delta_{\hat{k}}$ (*t*_2_*,t*_3_) using the main ADNI dataset, and applied them to the AIBL cohort. The predicted rates in AIBL were then used to assess MCI/Dementia progression prediction. In the CN group, 13 individuals progressed from CN to MCI/dementia, while 45 remained stable. Due to the limited number of participants in the MCI group (*n* = 8) with available follow-up data extending five years beyond the current visit (i.e., the 18-month assessment), prediction of MCI-to-dementia progression was not conducted in the AIBL cohort. Lastly, we selected a subset of ADNI participants who were excluded from the main experiments due to missing 48-month data. However, since these individuals had both 0- and 24-month follow-up visits along with sufficient clinical follow-up information, they were eligible for inclusion in the progression prediction analysis. We refer to this subset as the ADNI-external test set. Within this subset, 25 individuals in the CN group progressed to MCI/dementia, while 56 remained stable, but the mean age of these two groups was highly different. In the MCI group, 46 individuals progressed to dementia, whereas 43 remained unchanged in their clinical status, see Table 3.

## Results

### Prediction of atrophy/enlargement: Cross-validation with ADNI

**Figure 2.**
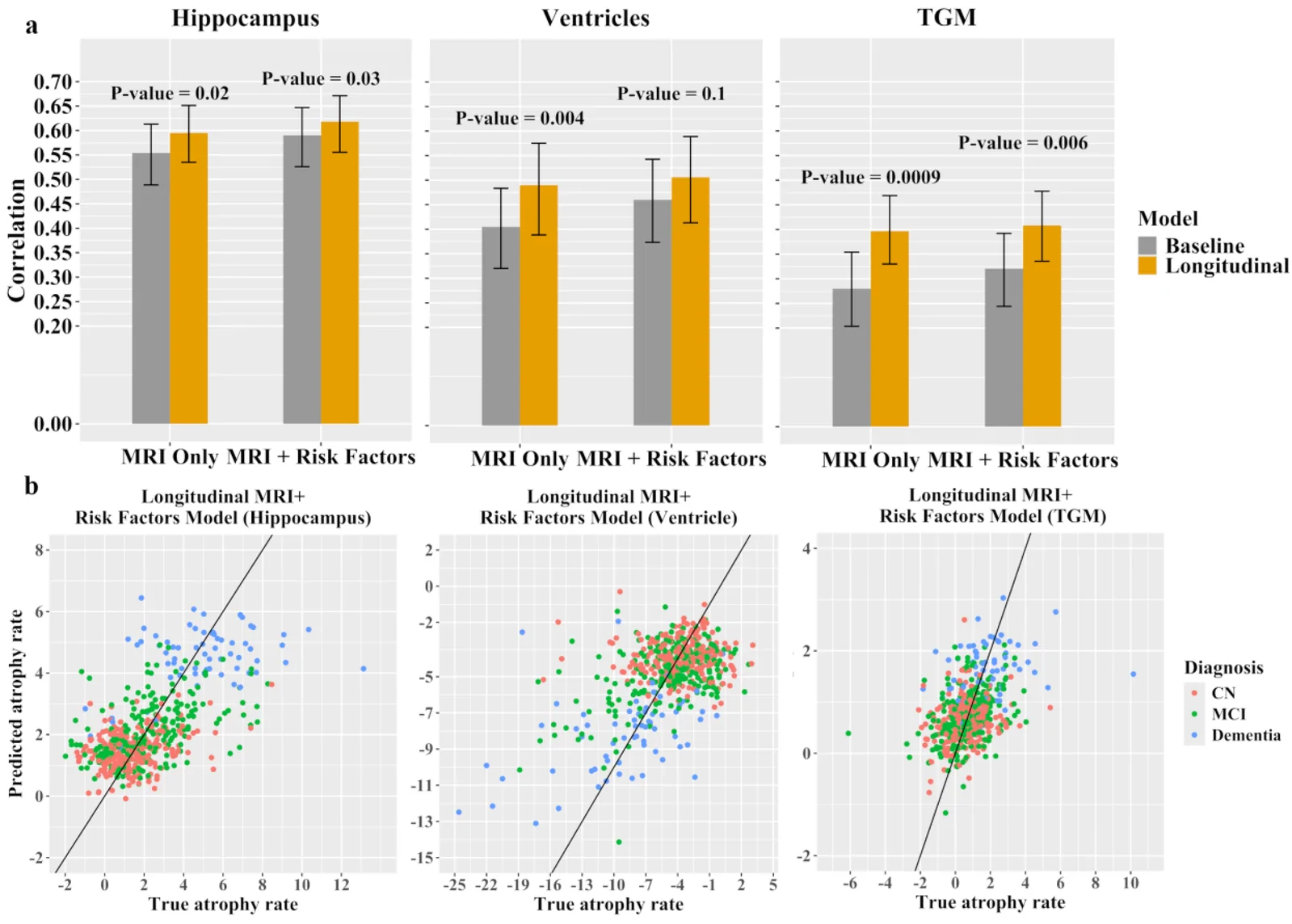
Prediction of annualized percentage change of volumes of different brain regions using 10-fold cross-validation on the main ADNI dataset. (a) Bar plots showing the correlation of different models based on baseline and longitudinal approaches. (b) Scatter plot illustrating the prediction of atrophy rates for the longitudinal approach of MRI + Risk Factors model, derived from a single computation run with median performance.

The validation results of all models are presented in Figure 2. Overall, our findings highlight that the longitudinal approach, particularly when incorporating additional risk factors, yielded the best performance for predicting annual atrophy/enlargement.

### Hippocampal atrophy

For the baseline approach utilizing only MRI data, the PCC for predicting the hippocampal atrophy rate was 0.55 (95% CI: 0.49, 0.61), with MAE of 1.36 (95% CI: 1.26, 1.46). Incorporating risk factors into the baseline MRI model, the PCC improved significantly to 0.59 (95% CI: 0.53, 0.65, p = 0.02), and the MAE decreased to 1.32 (95% CI: 1.22, 1.41). For the longitudinal approach using the MRI-only model, the PCC was 0.60 (95% CI: 0.54, 0.65), with an MAE of 1.31 (95% CI: 1.22, 1.41). When the longitudinal MRI model was combined with risk factors, the PCC significantly improved to 0.62 (95% CI: 0.56, 0.67, p = 0.04), with an estimated MAE of 1.29 (95% CI: 1.20, 1.38). In the MRI-only model, the PCC for the baseline approach significantly improved from 0.55 (95% CI: 0.49, 0.61) to 0.60 (95% CI: 0.54, 0.65) for the longitudinal approach (p = 0.02). Similarly, in the model incorporating both MRI and risk factors, the PCC increased from 0.59 (95% CI: 0.53, 0.65) in the baseline approach to 0.62 (95% CI: 0.56, 0.67) in the longitudinal approach, representing a significant improvement (p = 0.03).

### Ventricle enlargement

For predicting ventricle enlargement rate using the baseline approach relying solely on MRI data, the PCC was 0.40 (95% CI: 0.32, 0.48), with an MAE of 2.67 (95% CI: 2.48, 2.88). Incorporating risk factors into the baseline MRI model improved the PCC significantly to 0.46 (95% CI: 0.37, 0.54, p = 0.004), while reducing the MAE to 2.57 (95% CI: 2.38, 2.79). For the longitudinal approach using the MRI-only model, PCC increased to 0.49 (95% CI: 0.39, 0.58), and the MAE decreased to 2.52 (95% CI: 2.33, 2.72). The best performance was achieved with the longitudinal approach combining the MRI model and additional risk factors, yielding the PCC 0.51 (95% CI: 0.41, 0.59) and the MAE of 2.48 (95% CI: 2.29, 2.69), however, this improvement was not significant (p = 0.4) over the longitudinal MRI-only model. In order to compare the models using only MRI information for ventricle enlargement rate, the PCC for the baseline approach was 0.40 (95% CI: 0.32, 0.48). For the longitudinal approach, the PCC increased to 0.49 (95% CI: 0.39, 0.58), representing a significant improvement (p = 0.004). For the model incorporating both MRI and risk factor information, there was no significant improvement, with the PCC increasing from 0.46 (95% CI: 0.37, 0.54) in the baseline approach to 0.51 (95% CI: 0.41,0.59) in the longitudinal approach (p = 0.1).

### Gray matter atrophy

To predict the TGM atrophy, based on baseline approach utilizing only MRI data, the PCC was 0.28 (95% CI: 0.20, 0.35), with a MAE of 0.90 (95% CI: 0.83, 0.97). Incorporating risk factors into the MRI model improved the PCC to 0.32 (95% CI: 0.24, 0.39) and slightly reduced the MAE to 0.89 (95% CI: 0.82, 0.95), the difference between the two models was not significant (p = 0.1). For the longitudinal approach using only the MRI model, the PCC increased further to 0.40 (95% CI: 0.33, 0.47), with the MAE of 0.86 (95% CI: 0.80, 0.93). The longitudinal MRI + Risk factors model achieved a PCC of 0.41(95% CI: 0.34, 0.48) and an MAE of 0.86 (95% CI: 0.80, 0.93). The improvement over longitudinal MRI-only model was not significant (p = 0.6). In the MRI-only model, the PCC was 0.28 (95% CI: 0.20, 0.35) for the baseline approach and increased to 0.40 (95% CI: 0.33, 0.47) for the longitudinal approach, demonstrating a significant improvement (p = 0.0009). Furthermore, after incorporating risk factors alongside MRI information, we observed a significant improvement from 0.32 (95% CI: 0.24,0.39) in the baseline approach to 0.41 (95% CI: 0.34, 0.48) in the longitudinal approach (p = 0.006).

### Predictor importance

**Figure 3.**
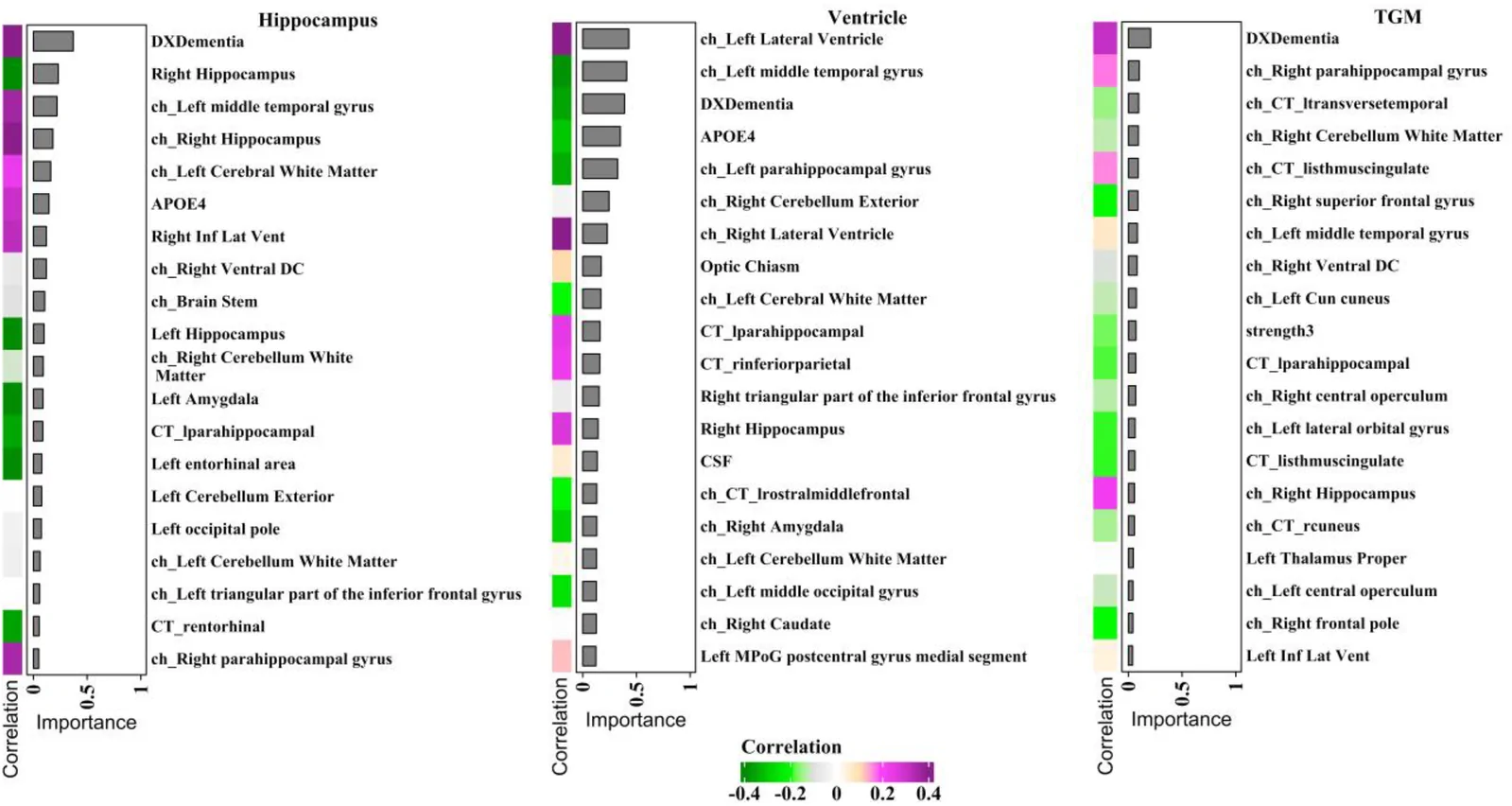
Importance of predictors across 10 runs of 10-fold CV for the longitudinal, MRI + risk factors model. Each heatmap consists of a separate single-column heatmap showing the correlation scores between each variable and the target label. Additionally, a bar graph illustrates the importance of each predictor, calculated as the mean of absolute values of regression coefficients across the cross-validation folds. Note that the regression coefficients are computed for standardized models. For brain imaging predictors, “ch” indicates change and “CT” indicates cortical thickness. Predictor names without “CT” refer to volumes. “DX” refers to the diagnosis at 24 months, defined across three columns: “DXCN”, “DX-Dementia”, and “DX-MCI”. Strength refers to variable coding of the field strengths of the scanners used to acquire the scans.

The most important predictors for atrophy/enlargement rates in the longitudinal MRI + risk factor model are displayed in Figure 3. For predicting hippocampal atrophy, the five most important predictors were dementia diagnosis, right hippocampal volume, and changes in volumes of left middle temporal gyrus, right hippocampus and left cerebral white matter. Both present day volumes and changes in volumes of various brain structures contributed to models. However, only present day cortical thickness values (and not the rate of change in cortical thickness) were included as top predictors. From risk factors, only dementia diagnosis and the number of *APOE4* alleles were included in the top predictors. Unsurprisingly, only the left or right (but not both) structure was included in the top predictors, with the exception of hippocampus volume. For predicting ventricle enlargement, the five most important predictors were changes in volumes of left lateral ventricle, left middle temporal gyrus, and left parahippocampal gyrus, dementia diagnosis, and the number of *APOE4* alleles. The change rates of volumes of various structures appeared more often in the top predictors than the present day volumes of structures. Few cortical thickness measures were included as well as change rate of left rostral middle frontal cortex. Again, from risk factors, only dementia diagnosis and the number of *APOE4* alleles were included in the top predictors. For TGM atrophy, the five most important predictors were dementia diagnosis, change rates of right parahippocampal gyrus volume, left transverse temporal cortical thickness, right cerebellum white matter volume, and left isthmus cortical thickness. Again, the change rates of volumes of various structures appeared more often in the top predictors than the present day volumes of structures. Both changes of and present day cortical thicknesses were included as top 20 predictors. The only risk factor in the top predictor list was the dementia diagnosis. For TGM, one of variables coding the MRI field strength entered to the model (strength3, indicating that all the measurements were done with 3.0 T scanner).

### Prediction of atrophy/enlargement: external evaluation with AIBL

**Figure 4.**
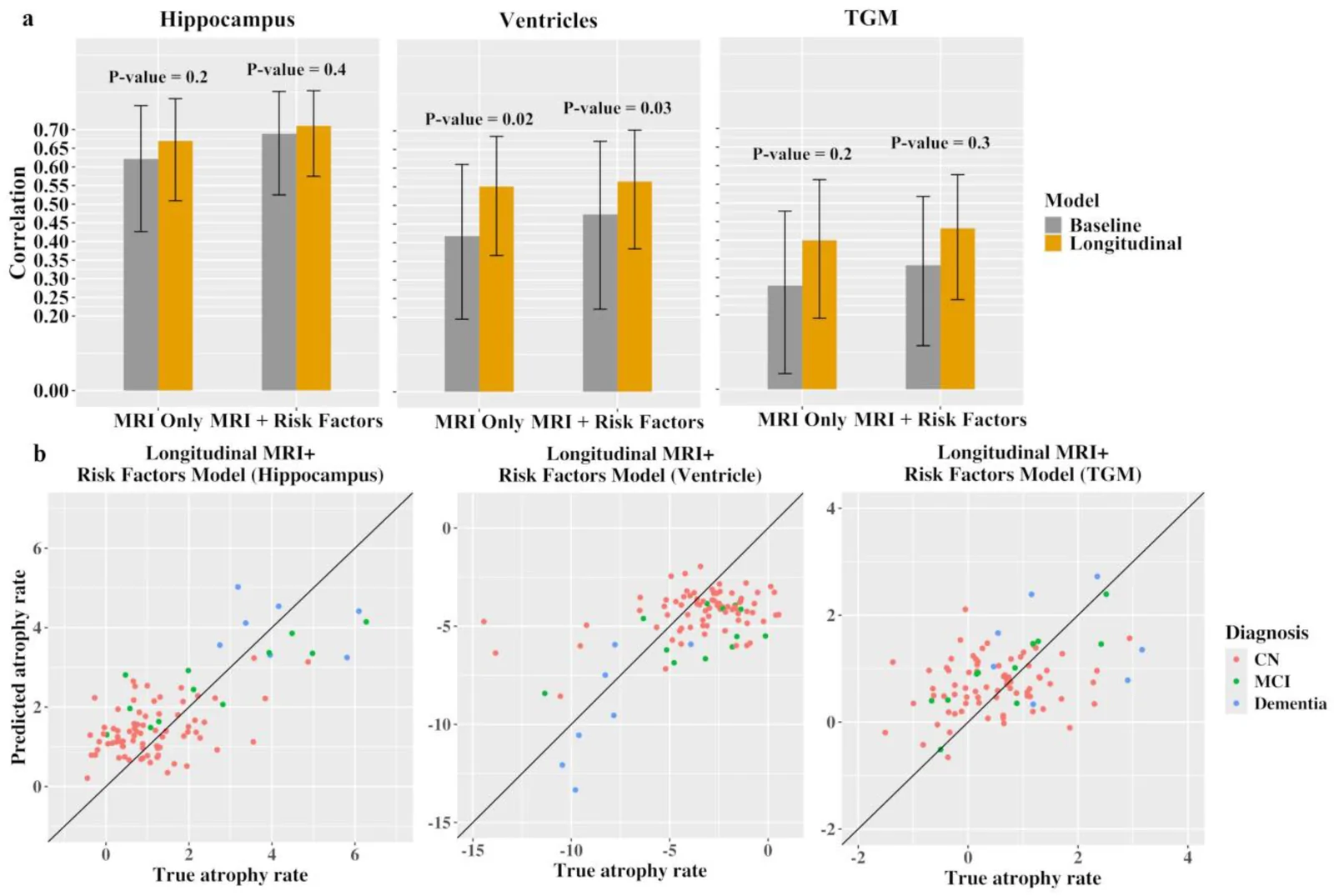
Prediction of annualized percentage change of volumes in different brain regions on the AIBL (test) dataset. (a) Bar plots showing the correlation scores of different models based on baseline and longitudinal approaches. (b) Scatter plots illustrating the prediction of atrophy rates for the longitudinal approach of MRI + Risk Factors model, derived from a single computation run with median performance.

Figure 4 presents the external evaluation results for atrophy/enlargement prediction using the AIBL dataset. These results demonstrate that the longitudinal approach outperforms the baseline approach when tested on an external dataset.

The external evaluation for hippocampal atrophy prediction using the baseline approach with the MRI only model resulted in a PCC of 0.62 (95% CI: 0.43, 0.76) and a MAE of 1.00 (95% CI: 0.87, 1.15). When applying the longitudinal approach and incorporating risk factors alongside MRI data, the PCC improved to 0.71 (95% CI: 0.57, 0.80), while the MAE decreased to 0.89 (95% CI: 0.78,1.01). The external evaluation for ventricle enlargement prediction using the baseline approach, which relied solely on MRI data, resulted in the PCC of 0.42 (95% CI: 0.19, 0.61) and the MAE of 2.40 (95% CI: 2.05, 2.79). In comparison, the longitudinal approach that combined MRI data with risk factor information achieved a higher PCC of 0.56 (95% CI: 0.38, 0.70) and a lower MAE of 2.14 (95% CI:1.83, 2.46). The external evaluation for TGM atrophy prediction using the baseline approach, which relied solely on MRI data, resulted in the PCC of 0.28 (95% CI: 0.04, 0.48) and the MAE of 0.82 (95% CI: 0.69,0.96). In comparison, the longitudinal approach that combined MRI data with risk factor information achieved a higher PCC of 0.43 (95% CI: 0.24, 0.58) and a slightly lower MAE of 0.78(95% CI: 0.66,0.90).

### Prediction of MCI/dementia progression

**Figure 5.**
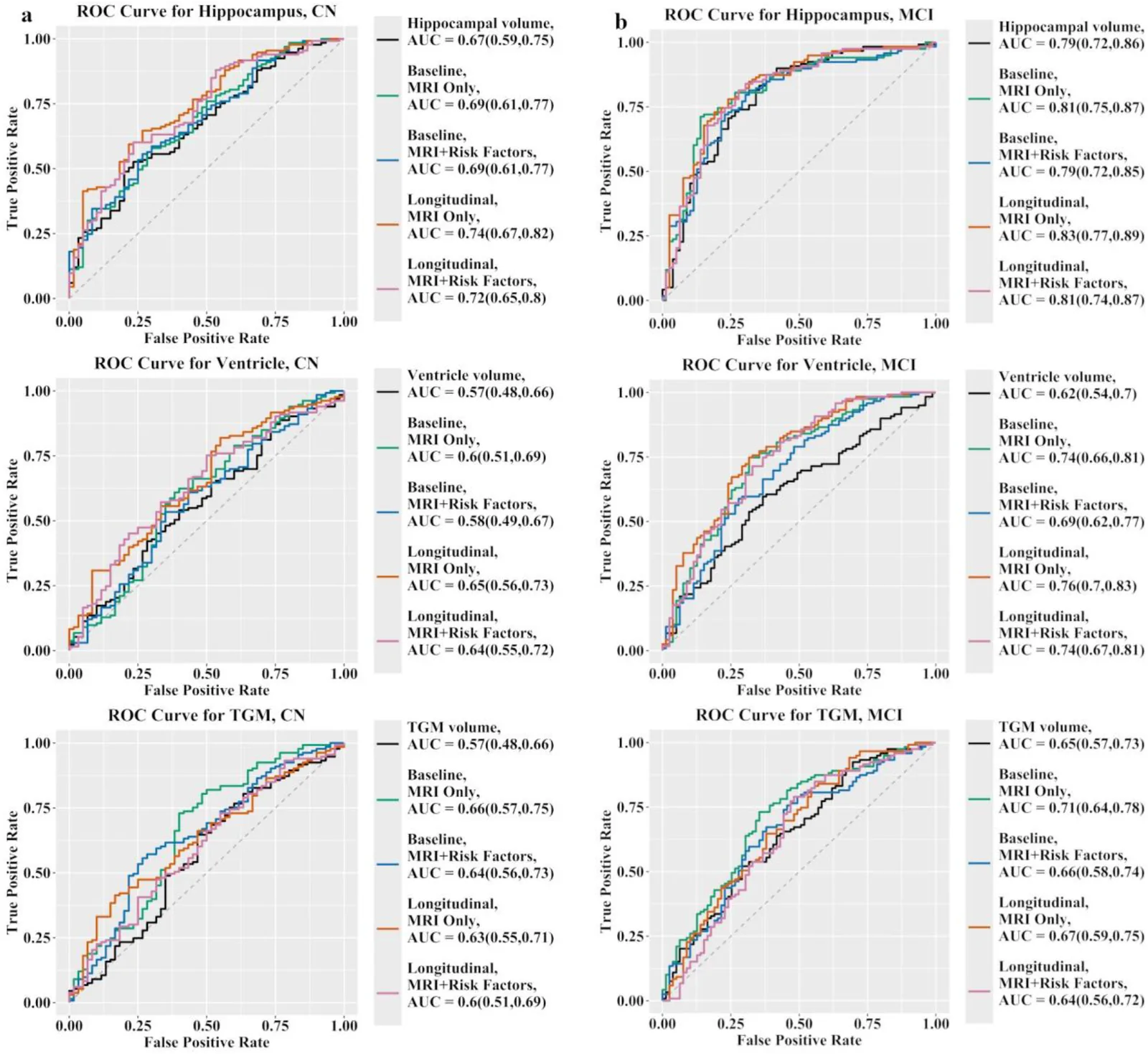
ROC curves of clinical status progression prediction in (a) CN and (b) MCI groups with different models and brain regions using ADNI-main dataset.

The ROC curves assessing performance of the predicted atrophy/enlargement rates in clinical status progression prediction are presented in Figure 5 for ADNI main dataset and in Supplemental Figures 1 and 2 for AIBL and ADNI external datasets. All AUC statistics are collected in Table 4. Overall, the clinical status progression prediction results highlight that a) predicted atrophy yielded better than chance progression predictions and the predicted atrophy scores typically reached higher AUCs than the corresponding present day volume information.

**Table 4.** AUCs for different models for clinical status progression prediction across three databases.

Brain regions	Models	Datasets				
		ADNI-main		AIBL	ADNI-external	
		CN-to-MCI	MCI-to-Dementia	CN-to-MCI	CN-to-MCI	MCI-to-Dementia
Hippocampus	Hippocampal volume	0.67 (0.59, 0.75)	0.79 (0.72, 0.86)	0.62 (0.42, 0.82)	0.69 (0.56, 0.82)	0.78 (0.69, 0.88)
	Baseline, MRI only	0.69 (0.61, 0.77)	0.81 (0.75, 0.87)	0.77 (0.62, 0.92)	0.73 (0.61, 0.84)	0.73 (0.62, 0.83)
	Baseline, MRI + Risk factors	0.69 (0.61, 0.77)	0.79 (0.72, 0.85)	0.84 (0.72, 0.96)	0.81 (0.71, 0.90)	0.83 (0.75, 0.91)
	Longitudinal, MRI only	0.74 (0.67, 0.82)	0.83 (0.77, 0.89)	0.84 (0.72, 0.95)	0.83 (0.72, 0.93)	0.85 (0.77, 0.93)
	Longitudinal, MRI + Risk factors	0.72 (0.65, 0.80)	0.81 (0.74, 0.87)	0.87 (0.76, 0.98)	0.83 (0.72, 0.93)	0.86 (0.79, 0.94)
Ventricles	Ventricle volume	0.57 (0.48, 0.66)	0.62 (0.54, 0.70)	0.65 (0.49, 0.81)	0.77 (0.66, 0.88)	0.61 (0.49, 0.73)
	Baseline, MRI only	0.60 (0.51, 0.69)	0.74 (0.66, 0.81)	0.66 (0.46, 0.87)	0.59 (0.45, 0.73)	0.70 (0.59, 0.81)
	Baseline, MRI + Risk factors	0.58 (0.49, 0.67)	0.69 (0.62, 0.77)	0.70 (0.49, 0.92)	0.56 (0.42, 0.70)	0.65 (0.54, 0.77)
	Longitudinal, MRI only	0.65 (0.56, 0.73)	0.76 (0.70, 0.83)	0.82 (0.66, 0.97)	0.69 (0.56, 0.81)	0.76 (0.66, 0.86)
	Longitudinal, MRI + Risk factors	0.64 (0.55, 0.72)	0.74 (0.67, 0.83)	0.83 (0.67, 0.99)	0.67 (0.54, 0.80)	0.75 (0.64, 0.85)
TGM	TGM volume	0.57 (0.48, 0.66)	0.65 (0.57, 0.73)	0.56 (0.34, 0.77)	0.49 (0.35, 0.62)	0.58 (0.46, 0.70)
	Baseline, MRI only	0.66 (0.57, 0.75)	0.71 (0.64, 0.78)	0.74 (0.57, 0.91)	0.57 (0.43, 0.71)	0.55 (0.42, 0.67)
	Baseline, MRI + Risk factors	0.64 (0.56, 0.73)	0.66 (0.58, 0.74)	0.74 (0.58, 0.90)	0.48 (0.34, 0.62)	0.56 (0.44, 0.69)
	Longitudinal, MRI only	0.63 (0.55, 0.71)	0.67 (0.59, 0.75)	0.74 (0.57, 0.90)	0.62 (0.50, 0.75)	0.55 (0.43, 0.67)
	Longitudinal, MRI + Risk factors	0.60 (0.51, 0.69)	0.64 (0.56, 0.72)	0.69 (0.51, 0.88)	0.60 (0.47, 0.73)	0.59 (0.46, 0.71)

### ADNI-main cohort

For the ADNI-Main cohort, using present day hippocampus volume achieved an AUC of 0.67 (95% CI: 0.59, 0.75) for the CN-to-MCI progression prediction. The predicted hippocampal atrophy (with longitudinal MRI + risk factors model) resulted in the improved progression prediction performance, yielding an AUC of 0.72 (95% CI: 0.65, 0.80). However, this improvement was not statistically significant (p = 0.10, DeLong test^52^). Similarly, the present day ventricle volume produced an AUC of 0.57 for CN-to-MCI progression prediction (95% CI:0.48, 0.66), which increased to 0.64 (95% CI:0.55,0.72) with the predicted ventricle enlargement (longitudinal MRI + risk factors model). This improvement was not statistically significant (p = 0.30, DeLong test). The present day TGM volume predicted CN-to-MCI progression with an AUC of 0.57 (95% CI: 0.48, 0.66), which increased to 0.60 (95% CI: 0.51, 0.69) with the predicted TGM atrophy (longitudinal MRI + risk factors). This increment was not statistically significant (p = 0.60).

Within MCI group, present day hippocampus volume predicted progression to dementia with an AUC of 0.79 (95% CI: 0.72, 0.86). Predicted atrophy (longitudinal MRI + risk factors model) did not significantly improve the performance (p = 0.50), yielding an AUC of 0.81 (95% CI: 0.74, 0.87). For ventricle volume, the AUC was 0.62 (95% CI: 0.54, 0.70), which significantly improved to 0.74 (95% CI: 0.67, 0.81) when predicting progression to dementia using predicted atrophy (longitudinal MRI + risk factors model, p = 0.04). For the TGM volume, the AUC for the TGM volume was 0.65 (95% CI: 0.57, 0.73) and predicted atrophy for longitudinal MRI + risk factors model yielded a similar AUC of 0.64 (95% CI:0.56, 0.72) (p = 0.9).

The comparison between different atrophy prediction models (baseline vs. longitudinal, MRI only versus MRI + risk factors) did not yield any clearly best model, but all the models performed similarly in the progression prediction.

### ADNI-external cohort

For the ADNI-external cohort among the CN group, present day hippocampus volume achieved an AUC of 0.69 (95% CI: 0.56, 0.82) for the CN-to-MCI progression prediction. The predicted hippocampal atrophy (with longitudinal MRI + risk factors model) resulted in the increased progression prediction performance, yielding an AUC of 0.83 (95% CI: 0.72,0.93). This improvement was statistically significant (p = 0.04, DeLong test). Similarly, the present day ventricle volume produced an AUC of 0.77 for CN-to-MCI progression prediction (95% CI:0.66, 0.88), which reduced to 0.67 (95% CI:0.54,0.80) with the predicted ventricle enlargement (longitudinal MRI + risk factors model). This reduction was not statistically significant (p = 0.2, DeLong test) and we hypothesize that the high AUC for ventricle volume based prediction was driven by a large age difference between the stable and progressive groups (see Table 3). The present day TGM volume predicted CN-to-MCI progression with an AUC of 0.49 (95% CI: 0.35, 0.62), which increased to 0.60 (95% CI: 0.47, 0.73) with the predicted TGM atrophy (longitudinal MRI + risk factors). This increment was not statistically significant (p = 0.2).

For the MCI group in the ADNI-external, the model using hippocampal volume alone achieved an AUC of 0.78 (95% CI: 0.69, 0.88). With the predicted hippocampal atrophy (longitudinal MRI + risk factors model) the progression prediction performance improved significantly, yielding an AUC of 0.86 (95% CI: 0.79, 0.94) (p = 0.03). The ventricle volume model achieved an AUC of 0.61 (95% CI: 0.49,0.73), which increased to 0.75 (95% CI: 0.64,0.85) with the predicted ventricle enlargement (longitudinal MRI + risk factors mode), but the increase was not significant (p = 0.1). The model using TGM volume resulted in an AUC of 0.58 (95% CI: 0.46,0.70), while the predicted TGM atrophy (longitudinal MRI + risk factors model) resulted to a similar AUC to 0.59 (95% CI: 0.46, 0.71) (p = 0.9).

Notably, the AUCs for clinical status progression prediction were consistently higher for atrophy scores predicted based on longitudinal model than for the atrophy scores based on baseline model. Addition of risk factors to the atrophy score prediction did not have much influence on the progression prediction, with the exception of the hippocampus and the baseline model, where atrophy scores predicted based on MRI + risk factors model provided better progression predictions than those based on MRI only model.

### AIBL cohort

Using hippocampus volume, we could predict the progression from CN to MCI with an AUC of 0.62 (95% CI: 0.42, 0.82). Using predicted atrophy scores significantly improved the prediction performance, resulting in an AUC of 0.87 (95% CI: 0.76, 0.98) (p = 0.02), highlighting the enhanced predictive value of this approach. The ventricle volume achieved an AUC of 0.65 (95% CI: 0.49, 0.81) while the AUC with predicted ventricle enlargement improved to 0.83 (95% CI: 0.67, 0.99), however, the improvement was not significant (p = 0.1). Using TGM volume, the AUC was 0.56 (95% CI: 0.34, 0.77) while the AUC with predicted TGM was increased to 0.69 (95% CI: 0.51, 0.88) (p = 0.3).

Here, the longitudinal MRI + risk factors model was clearly superior to the baseline MRI only model for progression prediction using predicted hippocampal atrophy and ventricle enlargement. With TGM, all the four models performed similarly in terms of progression prediction.

## Discussion

Hippocampal atrophy, ventricular enlargement, and TGM atrophy are three key MRI biomarkers commonly associated with AD progression and neurodegeneration.^53,54^ More recent randomized controlled trials, such as the LipiDiDiet studies, have further demonstrated their use as secondary endpoints, with reported effects on brain atrophy progression.^20,21^ In particular, hippocampal volume has been qualified as an enrichment biomarker for pre-dementia AD trials.^15^ Longitudinal changes in these MRI measures may also provide useful endpoints for evaluating treatment effects.^13^

This study aimed to predict the annual change in the hippocampus, ventricles, and total gray matter volumes (used as a proxy for whole-brain atrophy), in individuals with varying levels of cognitive impairment, ranging from healthy to dementia. To achieve this, we developed an ML model based on ENLR. We explored two approaches: (1) a baseline method relying on single-time-point data and (2) a longitudinal method incorporating information from two years prior to baseline. Both approaches were tested using models that utilized only MRI data and models that combined MRI data with additional risk factors, including age, sex, and baseline diagnosis. Our analysis also explored how incorporating longitudinal data affected the performance of these models.

The predictions for all three MRI markers were clearly better than the baseline prediction of no chance that would have the PCC of zero. Based on the PCC values, the hippocampal atrophy could be best predicted, followed by the ventricle enlargement, while the TGM atrophy was the most challenging to predict. Indeed, with the external test data (AIBL) hippocampal atrophy prediction had PCC of 0.71 while TGM atrophy prediction only reached PCC of 0.43.

Our findings showed that incorporating additional information into the baseline MRI model significantly enhanced its ability to predict hippocampal atrophy and ventricular enlargement. In our previous works, we have demonstrated the advantages of combining multiple data modalities—such as MRI and cognitive assessments — for prediction tasks relevant to dementia management.^28,38,55^ Also, other studies have emphasized the importance of integrating diverse data sources for enhanced predictive accuracy.^26,56,57^ Our current results align with these findings, but with a notable difference in the methodology. Instead of using baseline cognitive assessments as predictors, we utilized baseline diagnoses as predictors. Baseline diagnosis serves as a summary of cognitive assessments, encapsulating an individual's cognitive status. Using a single summary variable reduced data dimensionality and redundancy, as many cognitive measures convey overlapping information.

Incorporating data from two years prior to the current visit into a longitudinal model consistently enhanced predictive performance even if the performance improvement was in relatively modest especially in hippocampal atrophy prediction. With ADNI, the improvement was statistically significant when comparing baseline versus longitudinal using MRI-only models across all cases, including hippocampal atrophy, TGM atrophy, and ventricular enlargement. For MRI + risk factor models, adding historical data also improved performance, but the improvement was smaller compared to the MRIonly models. The improvement remained statistically significant for hippocampal and TGM atrophy but not for ventricular enlargement. Overall, the best predictions were achieved using the longitudinal MRI + risk factor model across all three brain regions. External validation using the AIBL dataset confirmed these findings, demonstrating that longitudinal models consistently provided more accurate predictions for all three types of annual change. Our findings extend those of earlier studies that have shown the importance of considering longitudinal data in prediction tasks based on brain imaging data.^58,59^ We speculate that the greater improvement observed in the MRI-only model compared to the MRI + risk factor model may be due to the absence of cognitive status information in MRI-only models. Adding historical data to baseline MRI data provides additional insights into individual structural changes over time, as the differences between past and baseline MRI scans vary across diagnostic groups (CN, MCI, AD). This temporal information helps the model differentiate individuals more effectively. In contrast, the MRI + risk factor model already includes diagnostic group information, which partially accounts for cognitive status, thereby reducing the relative contribution of historical data.

In longitudinal models, we investigated the importance of various predictors, including risk factors, MRI features and their annualized percentage changes between baseline and two years prior. A closer examination of the coefficient values in the longitudinal model revealed that baseline diagnosis is one of the most important features for predicting the annual percentage change in all three studied markers. Among the top selected features, APOE4 emerged as a significant predictor for hippocampal atrophy and ventricular enlargement, but not for TGM atrophy. Interestingly, many longitudinal MRI features were identified as important within the top selected features, emphasizing the value of incorporating longitudinal information into the models. Another notable observation is that, for predicting the annual percentage change in each biomarker, multiple brain regions contribute to the prediction task, rather than the respective region alone holding the highest importance. Among the top selected MRI features for predicting hippocampal atrophy and ventricular enlargement were the hippocampus, middle temporal gyrus, inferior lateral ventricles, and amygdala, all of which are highly relevant in AD progression.^53^ However, the top selected features for predicting TGM atrophy differed slightly from those for hippocampal and ventricular predictions and were not as strongly associated with AD. These results align with our expectations, as the hippocampus and ventricles are the primary brain regions affected by AD, even in the early stages of disease. In contrast, TGM represents the whole brain and exhibits a weaker association with AD compared to the hippocampus and ventricles. Overall, our findings indicate that multiple brain regions, along with their longitudinal information and associated risk factors, play a crucial role in predicting the annual percentage change in all these biomarkers.

The MRI scans in the ADNI dataset were drawn from different ADNI phases, and MRI data was acquired using different field strengths. This variability posed a challenge,^60,61^ particularly as our analysis considered multiple time points for each participant, two time points (current day and future) for baseline models, and three time points (past, current day, and future) for longitudinal models. For many participants, the field strength was not consistent across all time points. To address this issue, we introduced a new categorical variable representing the field strength at each time point and included it as a predictor in our models (see^37^). By doing so, we accounted for the variability in field strength and ensured it was considered in the analysis, reducing its potential impact on the model's performance. To streamline our analysis, we opted for a cross-sectional pipeline for MRI preprocessing using CAT12, prioritizing its simplicity and efficiency in reducing processing time. Our preliminary analyses revealed no significant performance differences between the cross-sectional and longitudinal preprocessing approaches for atrophy prediction in CAT12. Consequently, the cross-sectional pipeline was selected as a practical and effective choice for our study and the finding that longitudinal predictors outperformed cross-sectional ones supports this choice. However, the optimal approach for addressing the construction of the longitudinal predictors should be addressed systematically in future studies.

Although hippocampal and TGM atrophy, as well as ventricular enlargement, are critical factors in dementia progression, predicting volume changes in these regions has not been extensively studied. In particular, limited research has evaluated all three regions within the same dataset and pipeline to assess their roles in dementia progression. While various studies have investigated the associations of these biomarkers with progression to dementia,^54,62,63^ the prediction of changes over time and their relationship with progression to MCI or dementia remains underexplored. The prediction of total ventricular volume has been previously examined in the TADPOLE challenge,^10^ where the tasks were predicting clinical diagnosis, ADAS-Cog13 scores, and total ventricular volume. Another study by Liedes et al.^26^ focused on predicting the annualized rate of hippocampal atrophy using multivariate ML algorithms on the ADNI dataset. Our study shares similarities with Liedes et al.^26^ in predicting annualized hippocampal atrophy but differs in three key ways: (1) we analyzed all three regions—hippocampus, TGM, and ventricles—together within the same dataset and workflow, rather than focusing solely on hippocampal atrophy; (2) we developed a longitudinal model that incorporates data from prior time points, enabling us to investigate the impact of longitudinal predictors on the performance of our predictive models; and (3) we evaluated the ability of our models to predict progression to MCI or dementia in both healthy and MCI groups.

We assessed the effectiveness of our predictive models in estimating the annual percentage changes in hippocampal, TGM, and ventricular volumes to predict dementia progression in both healthy and MCI groups. Results from the main ADNI dataset showed that predicted hippocampal atrophy and ventricular enlargement outperformed actual biomarker volumes in predicting progression to MCI or dementia in both CN and MCI groups. The most notable improvements came from predicted hippocampal atrophy, particularly using longitudinal approaches. For CN individuals, the predictive accuracy for progression to MCI/dementia improved from an AUC of 0.67 (using actual hippocampal volume) to 0.73 (using predicted atrophy with the longitudinal method). Similarly, in MCI individuals, the AUC for predicting progression to dementia increased from 0.79 to 0.83. However, no such improvement was observed for TGM atrophy, as predicted TGM atrophy did not provide better predictions compared to actual TGM volume and AUCs remained mostly below 0.70. Results from the external validation datasets, (AIBL and ADNI-external) showed similar trends. While our models were not specifically designed to predict progression to MCI or dementia, the predicted annual percentage changes—especially for hippocampal atrophy—performed well and the performance was comparable to previous MRI-based approaches for predicting dementia progression in CN individuals.^64,65^ This analysis demonstrates that predicted future atrophy scores can function as single valued and easily interpretable indexes of potential future cognitive decline. Also, they might be applicable when selecting/stratifying population for clinical trials, particularly when combined with disease-specific biomarkers.

In summary, our study highlights the importance of incorporating longitudinal data to improve predictions of annual changes in key biomarkers linked to dementia. By integrating MRI data and risk factors into machine learning models, we significantly enhanced predictive accuracy, particularly for hippocampal atrophy and ventricular enlargement. The inclusion of longitudinal data allowed us to capture individual structural changes over time, improving the model's ability to differentiate between diagnostic groups. These findings emphasize the advantages of combining diverse data sources and tracks brain changes over time to better predict dementia progression. Future research should further explore the integration of multiple data modalities and assess the long-term impact of longitudinal data to refine predictive models for dementia.

## Supplemental Material

sj-pdf-1-alz-10.1177_13872877261471049 - Supplemental material for Predicting future brain atrophy based on longitudinal MRI Supplemental material, sj-pdf-1-alz-10.1177_13872877261471049 for Predicting future brain atrophy based on longitudinal MRI by Maryam Hadji, Elaheh Moradi and Jussi Tohka in Journal of Alzheimer's Disease

## References

[1] Nichols E Vos T . The estimation of the global prevalence of dementia from 1990-2019 and forecasted prevalence through 2050: an analysis for the global burden of disease (GBD) study 2019. Alzheimers Dement 2021; 17: e051496.

[2] Lanctot KL Hahn-Pedersen JH Eichinger CS , et al. Burden of illness in people with Alzheimer’s disease: a systematic review of epidemiology, comorbidities and mortality. J Prev Alzheimers Dis 2024; 11: 97–107. 38230722 10.14283/jpad.2023.61 PMC10225771

[3] Colliot O . Machine learning for brain disorders. New York, NY: Humana Press, 2023. 37988480

[4] Ansart M Epelbaum S Bassignana G , et al. Predicting the progression of mild cognitive impairment using machine learning: a systematic, quantitative and critical review. Med Image Anal 2021; 67: 101848. 33091740 10.1016/j.media.2020.101848

[5] Frisoni GB Altomare D Ribaldi F , et al. Dementia prevention in memory clinics: recommendations from the European task force for brain health services. Lancet Reg Health Eur 2023; 26: 100576. 36895446 10.1016/j.lanepe.2022.100576 PMC9989648

[6] Reuben DB Kremen S Maust DT . Dementia prevention and treatment: a narrative review. JAMA Intern Med 2024; 184: 563–572. 38436963 10.1001/jamainternmed.2023.8522

[7] Kim H Lee S Levine A , et al. Novel measures of cognition and function for the AD spectrum in the novel measures for Alzheimer’s disease prevention trials (NoMAD) project: psychometric properties, convergent validation, and contrasts with established measures. Alzheimers Dement 2024; 20: 5089–5101. 38963127 10.1002/alz.13757 PMC11350053

[8] Grochowalski JH Liu Y Siedlecki KL . Examining the reliability of ADAS-cog change scores. Aging Neuropsychol Cogn 2016; 23: 513–529. 10.1080/13825585.2015.1127320 26708116

[9] Salthouse TA Nesselroade JR Berish DE . Short-term variability in cognitive performance and the calibration of longitudinal change. J Gerontol B Psychol Sci Soc Sci 2006; 61: P144–P151. 10.1093/geronb/61.3.p144 PMC3838959 16670183

[10] Marinescu RV Oxtoby NP Young AL , et al. The Alzheimer’s Disease Prediction of Longitudinal Evolution (TADPOLE) challenge: Results after 1 year follow-up. Mach Learn Biomed Imaging 2021; 1: 1–60.

[11] Prakash M Abdelaziz M Zhang L , et al. Quantitative longitudinal predictions of Alzheimer’s disease by multi-modal predictive learning. J Alzheimers Dis 2021; 79: 1533–1546. 33459714 10.3233/JAD-200906

[12] Jack CR Shiung MM Gunter JL , et al. Comparison of different MRI brain atrophy rate measures with clinical disease progression in AD. Neurology 2004; 62: 591–600. 14981176 10.1212/01.wnl.0000110315.26026.ef PMC2730165

[13] Cash DM Rohrer JD Ryan NS , et al. Imaging endpoints for clinical trials in Alzheimer’s disease. Alzheimers Res Ther 2014; 6: 87. 25621018 10.1186/s13195-014-0087-9 PMC4304258

[14] Hunter TR Santos LE Tovar-Moll F , et al. Alzheimer’s disease biomarkers and their current use in clinical research and practice. Mol Psychiatry 2025; 30: 272–284. 39232196 10.1038/s41380-024-02709-z

[15] Hill DL Schwarz AJ Isaac M , et al. Coalition against Major diseases/European medicines agency biomarker qualification of hippocampal volume for enrichment of clinical trials in predementia stages of Alzheimer’s disease. Alzheimers Dement 2014; 10: 421–429. 24985687 10.1016/j.jalz.2013.07.003

[16] Drago V Babiloni C Bartrés-Faz D , et al. Disease tracking markers for Alzheimer’s disease at the prodromal (MCI) stage. J Alzheimers Dis 2011; 26: 159–199. 21971460 10.3233/JAD-2011-0043

[17] Younes L Albert M Moghekar A , et al. Identifying changepoints in biomarkers during the preclinical phase of Alzheimer’s disease. Front Aging Neurosci 2019; 11: 74. 31001108 10.3389/fnagi.2019.00074 PMC6454004

[18] Bertens D Knol DL Scheltens P , et al. Temporal evolution of biomarkers and cognitive markers in the asymptomatic, MCI, and dementia stage of Alzheimer’s disease. Alzheimers Dement 2015; 11: 511–522. 25150730 10.1016/j.jalz.2014.05.1754 PMC4336230

[19] Nestor SM Rupsingh R Borrie M , et al. Ventricular enlargement as a possible measure of Alzheimer’s disease progression validated using the Alzheimer’s disease neuroimaging initiative database. Brain 2008; 131: 2443–2454. 18669512 10.1093/brain/awn146 PMC2724905

[20] Soininen H Solomon A Visser PJ , et al. 36-month Lipididiet multinutrient clinical trial in prodromal Alzheimer’s disease. Alzheimers Dement 2021; 17: 29–40. 32920957 10.1002/alz.12172 PMC7821311

[21] Soininen H Solomon A Visser PJ , et al. 24-month Intervention with a specific multinutrient in people with prodromal Alzheimer’s disease (LipiDiDiet): a randomised, double-blind, controlled trial. Lancet Neurol 2017; 16: 965–975. 29097166 10.1016/S1474-4422(17)30332-0 PMC5697936

[22] Dawe RJ Yu L Arfanakis K , et al. Late-life cognitive decline is associated with hippocampal volume, above and beyond its associations with traditional neuropathologic indices. Alzheimers Dement 2020; 16: 209–218. 31914231 10.1002/alz.12009 PMC6953608

[23] Apostolova LG Green AE Babakchanian S , et al. Hippocampal atrophy and ventricular enlargement in normal aging, mild cognitive impairment (MCI), and Alzheimer disease. Alzheimer Dis Assoc Disord 2012; 26: 17–27. 22343374 10.1097/WAD.0b013e3182163b62 PMC3286134

[24] Goukasian N Porat S Blanken A , et al. Cognitive correlates of hippocampal atrophy and ventricular enlargement in adults with or without mild cognitive impairment. Dement Geriatr Cogn Disord Extra 2019; 9: 281–293. 10.1159/000490044 PMC6751474 31572424

[25] Venkatraghavan V Bron EE Niessen WJ , et al. Disease progression timeline estimation for Alzheimer’s disease using discriminative event based modeling. Neuroimage 2019; 186: 518–532. 30471388 10.1016/j.neuroimage.2018.11.024

[26] Liedes H Lötjönen J Kortelainen JM , et al. Multivariate prediction of hippocampal atrophy in Alzheimer’s disease. J Alzheimers Dis 2019; 68: 1453–1468. 30909211 10.3233/JAD-180484

[27] Ellis KA Bush AI Darby D , et al. The Australian imaging, biomarkers and lifestyle (AIBL) study of aging: methodology and baseline characteristics of 1112 individuals recruited for a longitudinal study of Alzheimer’s disease. Int Psychogeriatr 2009; 21: 672–687. 19470201 10.1017/S1041610209009405

[28] Moradi E Dahnke R Imani V , et al. Integrating plasma, MRI, and cognitive biomarkers for personalized prediction of decline across cognitive domains. Neurobiol Aging 2025; 155: 53–65. 40712372 10.1016/j.neurobiolaging.2025.06.010

[29] Gaser C Dahnke R Thompson PM , et al. CAT: a computational anatomy toolbox for the analysis of structural MRI data. Gigascience 2024; 13: giae049. 10.1093/gigascience/giae049 PMC11299546 39102518

[30] Ashburner J Friston KJ . Diffeomorphic registration using geodesic shooting and Gauss–Newton optimisation. Neuroimage 2011; 55: 954–967. 21216294 10.1016/j.neuroimage.2010.12.049 PMC3221052

[31] Karaman BK Mormino EC Sabuncu MR , et al. Machine learning based multi-modal prediction of future decline toward Alzheimer’s disease: an empirical study. PLoS One 2022; 17: e0277322. 10.1371/journal.pone.0277322 PMC9668188 36383528

[32] Dahnke R Yotter RA Gaser C . Cortical thickness and central surface estimation. Neuroimage 2013; 65: 336–348. 23041529 10.1016/j.neuroimage.2012.09.050

[33] Yotter RA Thompson PM Gaser C . Algorithms to improve the reparameterization of spherical mappings of brain surface meshes. J Neuroimaging 2011; 21: e134–e147. 10.1111/j.1552-6569.2010.00484.x 20412393

[34] Desikan RS Ségonne F Fischl B , et al. An automated labeling system for subdividing the human cerebral cortex on MRI scans into gyral based regions of interest. Neuroimage 2006; 31: 968–980. 16530430 10.1016/j.neuroimage.2006.01.021

[35] Zou H Hastie T . Regularization and variable selection via the elastic net. J R Stat Soc Ser B Stat Methodol 2005; 67: 301–320.

[36] Tohka J Huttunen H . Alzheimer’s disease neuroimaging initiative. Comparison of feature selection techniques in machine learning for anatomical brain MRI in dementia. Neuroinformatics 2016; 14: 279–296. 26803769 10.1007/s12021-015-9292-3

[37] Linn KA Gaonkar B Doshi J , et al. Addressing confounding in predictive models with an application to neuroimaging. Int J Biostat 2016; 12: 31–44. 26641972 10.1515/ijb-2015-0030 PMC5154735

[38] Moradi E Prakash M Hall A , et al. Machine learning prediction of future amyloid beta positivity in amyloid-negative individuals. Alzheimers Res Ther 2024; 16: 46. 38414035 10.1186/s13195-024-01415-w PMC10900722

[39] Tohka J Van Gils M . Evaluation of machine learning algorithms for health and wellness applications: a tutorial. Comput Biol Med 2021; 132: 104324. 33774270 10.1016/j.compbiomed.2021.104324

[40] Hastie T Qian J Tay K . An introduction to glmnet. CRAN R Project. 2025.

[41] Hittner JB May K Silver NC . A Monte Carlo evaluation of tests for comparing dependent correlations. J Gen Psychol 2003; 130: 149–168. 12773018 10.1080/00221300309601282

[42] Dunn OJ Clark V . Correlation coefficients measured on the same individuals. J Am Stat Assoc 1969; 64: 366–377.

[43] Diedenhofen B Musch J . Cocor: a comprehensive solution for the statistical comparison of correlations. PloS One 2015; 10: e0121945. 10.1371/journal.pone.0121945 PMC4383486 25835001

[44] Lewis JD Evans AC Tohka J , et al. T1 white/gray contrast as a predictor of chronological age, and an index of cognitive performance. Neuroimage 2018; 173: 341–350. 29501876 10.1016/j.neuroimage.2018.02.050

[45] Johnson WE Li C Rabinovic A . Adjusting batch effects in microarray expression data using empirical Bayes methods. Biostatistics 2007; 8: 118–127. 16632515 10.1093/biostatistics/kxj037

[46] Fortin J-P Cullen N Sheline YI , et al. Harmonization of cortical thickness measurements across scanners and sites. Neuroimage 2018; 167: 104–120. 29155184 10.1016/j.neuroimage.2017.11.024 PMC5845848

[47] Kuhn M . Building predictive models in R using the caret package. J Stat Softw 2008; 28: 1–26.27774042

[48] Diedenhofen B . cocor: Comparing correlations. R package version 1.1-4. Vienna, Austria: Comprehensive R Archive Network (CRAN). https://CRAN.R-project.org/package=cocor (2022).

[49] Robin X Turck N Hainard A , et al. Package ‘pROC’. *Package “pROC*, http://download.nust.na/pub3/cran/web/packages/pROC/pROC.pdf (2021, accessed 28 May 2026).

[50] Wickham H . Ggplot2. Wiley Interdiscip Rev Comput Stat 2011; 3: 180–185.

[51] Gu Z Eils R Schlesner M . Complex heatmaps reveal patterns and correlations in multidimensional genomic data. Bioinformatics 2016; 32: 2847–2849. 27207943 10.1093/bioinformatics/btw313

[52] DeLong ER DeLong DM Clarke-Pearson DL . Comparing the areas under two or more correlated receiver operating characteristic curves: a nonparametric approach. Biometrics 1988: 837–845. 3203132

[53] Pini L Pievani M Bocchetta M , et al. Brain atrophy in Alzheimer’s disease and aging. Ageing Res Rev 2016; 30: 25–48. 26827786 10.1016/j.arr.2016.01.002

[54] Lee J Heo D Choi K-H , et al. Impact of the ventricle size on Alzheimer’s disease progression: a retrospective longitudinal study. Dement Neurocognitive Disord 2024; 23: 95. 10.12779/dnd.2024.23.2.95 PMC11073924 38720825

[55] Moradi E Pepe A Gaser C , et al. Machine learning framework for early MRI-based Alzheimer’s conversion prediction in MCI subjects. Neuroimage 2015; 104: 398–412. 25312773 10.1016/j.neuroimage.2014.10.002 PMC5957071

[56] Rahim N El-Sappagh S Ali S , et al. Prediction of Alzheimer’s progression based on multimodal deep-learning-based fusion and visual explainability of time-series data. Inf Fusion 2023; 92: 363–388.

[57] Reas ET Shadrin A Frei O , et al. Improved multimodal prediction of progression from MCI to Alzheimer’s disease combining genetics with quantitative brain MRI and cognitive measures. Alzheimers Dement 2023; 19: 5151–5158. 37132098 10.1002/alz.13112 PMC10620101

[58] Khundrakpam B Booij L Jeon S , et al. Individualized prediction of future cognition based on developmental changes in cortical anatomy. Neuroimage Rep 2022; 2: 100127. 40567557 10.1016/j.ynirp.2022.100127 PMC12172889

[59] Bhagwat N Viviano JD Voineskos AN , et al. Modeling and prediction of clinical symptom trajectories in Alzheimer’s disease using longitudinal data. PLoS Comput Biol 2018; 14: e1006376. 10.1371/journal.pcbi.1006376 PMC6157905 30216352

[60] Turrisi R Pati S Pioggia G , et al. Adapting to evolving MRI data: a transfer learning approach for Alzheimer’s disease prediction. Neuroimage 2025; 307: 121016. 39826774 10.1016/j.neuroimage.2025.121016

[61] Imani V Prakash M Zare M , et al. Comparison of single and multitask learning for predicting cognitive decline based on MRI data. IEEE Access 2021; 9: 154275–154291.

[62] Zhang B Xu M Wu Q , et al. Definition and analysis of gray matter atrophy subtypes in mild cognitive impairment based on data-driven methods. Front Aging Neurosci 2024; 16: 1328301. 38894849 10.3389/fnagi.2024.1328301 PMC11183285

[63] Sapkota S McFall GP Masellis M , et al. Differential cognitive decline in Alzheimer’s disease is predicted by changes in ventricular size but moderated by apolipoprotein E and pulse pressure. J Alzheimers Dis 2022; 85: 545–560. 34864669 10.3233/JAD-215068

[64] Strange BA Zhang L Sierra-Marcos A , et al. Predicting the future development of mild cognitive impairment in the cognitively healthy elderly. *bioRxiv* 2020. 10.1101/2020.07.30.227496 [Preprint]. Posted July 31, 2020.

[65] Achterberg HC Van Der Lijn F Den Heijer T , et al. Hippocampal shape is predictive for the development of dementia in a normal, elderly population. Hum Brain Mapp 2014; 35: 2359–2371. 24039001 10.1002/hbm.22333 PMC6869385

